# Differential Morpho-Physiological and Transcriptomic Responses to Heat Stress in Two Blueberry Species

**DOI:** 10.3390/ijms22052481

**Published:** 2021-03-01

**Authors:** Jodi Callwood, Kalpalatha Melmaiee, Krishnanand P. Kulkarni, Amaranatha R. Vennapusa, Diarra Aicha, Michael Moore, Nicholi Vorsa, Purushothaman Natarajan, Umesh K. Reddy, Sathya Elavarthi

**Affiliations:** 1 Department of Agriculture and Natural Resources, Delaware State University, Dover, DE 19901, USA; j.callwoo@gmail.com (J.C.); kkulkarni@desu.edu (K.P.K.); avennapusa@desu.edu (A.R.V.); daicha@desu.edu (D.A.); selavarthi@desu.edu (S.E.); 2 Optical Science Center for Applied Research (OSCAR), Delaware State University, Dover, DE 19901, USA; mmoore@desu.edu; 3 Philip E. Marucci Center for Blueberry and Cranberry Research and Extension, Rutgers University, Chatsworth, NJ 08019, USA; vorsa@njaes.rutgers.edu; 4 Department of Biology and Gus R. Douglass Institute, West Virginia State University, Institute, WV 25112, USA; pnatarajan@wvstateu.edu (P.N.); ureddy@wvstateu.edu (U.K.R.)

**Keywords:** blueberry, differentially expressed genes, heat stress, RNAseq, pathway analysis

## Abstract

Blueberries (*Vaccinium* spp.) are highly vulnerable to changing climatic conditions, especially increasing temperatures. To gain insight into mechanisms underpinning the response to heat stress, two blueberry species were subjected to heat stress for 6 and 9 h at 45 °C, and leaf samples were used to study the morpho-physiological and transcriptomic changes. As compared with *Vaccinium corymbosum*, *Vaccinium darrowii* exhibited thermal stress adaptation features such as small leaf size, parallel leaf orientation, waxy leaf coating, increased stomatal surface area, and stomatal closure. RNAseq analysis yielded ~135 million reads and identified 8305 differentially expressed genes (DEGs) during heat stress against the control samples. In *V. corymbosum*, 2861 and 4565 genes were differentially expressed at 6 and 9 h of heat stress, whereas in *V. darrowii*, 2516 and 3072 DEGs were differentially expressed at 6 and 9 h, respectively. Among the pathways, the protein processing in the endoplasmic reticulum (ER) was the highly enriched pathway in both the species: however, certain metabolic, fatty acid, photosynthesis-related, peroxisomal, and circadian rhythm pathways were enriched differently among the species. KEGG enrichment analysis of the DEGs revealed important biosynthesis and metabolic pathways crucial in response to heat stress. The GO terms enriched in both the species under heat stress were similar, but more DEGs were enriched for GO terms in *V. darrowii* than the *V. corymbosum*. Together, these results elucidate the differential response of morpho-physiological and molecular mechanisms used by both the blueberry species under heat stress, and help in understanding the complex mechanisms involved in heat stress tolerance.

## 1. Introduction

Blueberry (*Vaccinium* spp.), a North American native species domesticated in the 1950s, is globally popular for vitamin C and antioxidant concentrations, earning classification as a superfood for its positive health impacts [1,2,3,4,5,6]. In 2016, the United States was the largest blueberry producer, producing 690 million pounds of cultivated blueberries valued at around $700 million [3]. Global demand for blueberries has risen in recent years, as reflected in the export of 58 million pounds of blueberries from the United States in 2017 [7]. Canada was the second-largest producer of blueberries, with 109,007 tons of blueberries, followed by Poland, producing 12,731 tons, and Germany, producing 10,277 tons in 2016. Blueberry cultivation has been expanding in several countries, including China, India, and Mexico [8,9]. Overall, with increased consumer awareness of associated health benefits, blueberry is an economically important fruit crop globally.

Blueberries are highly vulnerable to changing environmental conditions and require precise chilling hours (200–1000 h), soil pH (<5.8), soil composition, and atmospheric temperature range (20–26 °C) for optimal plant growth and productivity [10,11,12,13]. Previous study reported adverse effects of climate change as the main reason for yield reductions in blueberry [13]. Other studies have also indicated that soil pH, chilling hours, and temperature range required for blueberry growth are more stringent [3,10,11,12,13]. Unpredictable weather patterns triggered by climate change causes extreme abiotic and biotic stresses, which increases the difficulty of growing blueberries. Due to global climate change effects, further research involving comprehensive phenotypic, molecular, and genetic characterization of the impact of heat stress on blueberries has become essential. Comparative transcriptome analyses performed in the past few years have revealed molecular and genetic components of several traits, including fruit and color development [14], flowering pathways [15], fruit ripening and biosynthesis of bioactive compounds [16], waxy coating on fruit [17], anthocyanin synthesis [18], and cold acclimation pathways [19] in blueberry. However, no studies have reported the transcriptome analysis of blueberry plants under high-temperature stress.

Many commercially available blueberry varieties exhibit variation in chilling hours and temperature tolerance based on their origin and genetic heritage [20]. *Vaccinium corymbosum,* a diploid highbush evergreen or deciduous shrub native to northern regions of North America, represents 75% of blueberry hybrids [1,10,21]. *Vaccinium darrowii,* a diploid evergreen shrub native to Florida and a southern highbush blueberry with chilling requirements < 600 h, has been combined with other blueberry species to generate varieties with reduced chilling hours and high-temperature survivability [1,11]. Such genetic combinations were highly successful in yielding hybrid berries specific to different temperature regions [1]. Moreover, many commercially available varieties have *V*. *corymbosum* and *V*. *darrowii* genetic background. Thus, examining divergent species from different native climates under heat stress conditions can help to understand the genetic responses and mechanisms involved in stress tolerance.

In the present study, we compared the response of *V. corymbosum* and *V. darrowii* plants to heat stress under controlled environmental conditions. Our study combined morpho-physiological observations and RNAseq analysis to profile mechanisms involved in their response to heat stress. The plants were exposed to 45 °C for 0, 6, and 9-h of heat stress, and leaf samples were used for microscopy analysis to understand the changes in stomatal function, stomatal internal organelle structures, and gene expression.

## 2. Results and Discussion

We investigated the impact of heat stress and whole-genome level transcriptome changes in *Vaccinium* species.

### 2.1. Morpho-Physiological Response to Heat Stress

The leaves in *V. darrowii* plants were clustered, oriented in multiple directions, and parallel to direct sunlight exposure in some cases. In contrast, *V. corymbosum* exhibited broader leaves with the perpendicular orientation to sunrays allowing maximum solar contact with leaf surface area (Figure S1A,B). Parallel leaf orientation is vital to prevent the dispersal of solar rays, thereby reduced high light interception and leaf temperature that assist in maintaining cellular functions under heat stress [22,23]. The orientation of leaves parallel to sun rays in *V. darrowii* indicated solar avoidance in heat-tolerant plants preventing overwhelming solar rays from directly impacting the leaf [21,24]. Moreover, *V. darrowii* leaves had a waxy coating and small leaf size (Figure S1B), which provides an additional mechanism to prevent water loss during high-temperature conditions [25].

**Figure 1:**
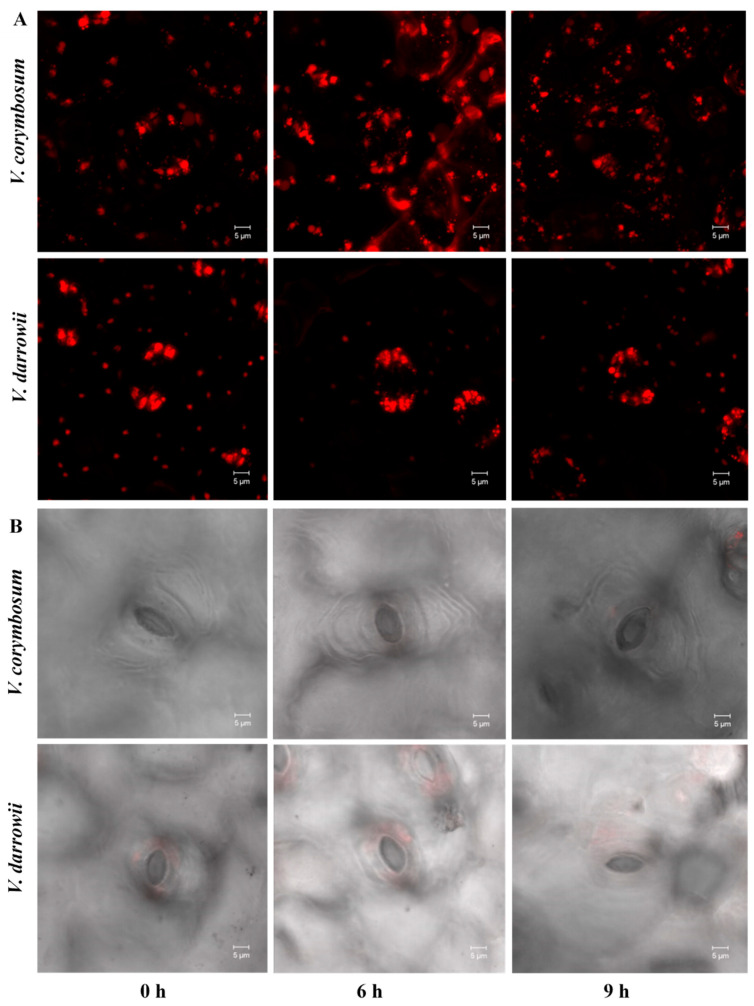
Laser scanning confocal microscopy (LSCM) imaging of stomata internal organelles and cell surface features of two blueberry species. (**A**) Stomata internal organelle surface area at different heat stress imposition periods (0, 6, and 9-h heat stress): Maximum projection of z stack with staining shows the wide opened structure of stoma organelle surface area that exhibits an intense red signal in *V. darrowii* compared to *V. corymbosum*. (**B**) LSCM micrographs showing the surface structure of stomata cell opening (guard cells).

Imaging analysis with laser scanning confocal microscopy revealed the effect of heat stress on stomata. Images of auto-fluorescent stomata internal organelles with a red auto-fluorescent signal showed no structural surface differences in *V. corymbosum* with increasing heat stress periods (6 and 9 h), but *V. darrowii* showed a difference in stomata organelle structures (Figure 1A). The average surface area of internal stomatal organelles did not show any discernable changes in *V. corymbosum* but expanded with increased heat stress in *V. darrowii* (Figure S1C). Previous studies have reported that plant-specific anatomical traits, such as reduced stomata surface area with shrinkage of cells and structural alterations in organelles, alter the stomatal pore (stomata opening). The surface area of stomatal cells helps maintain the water potential in tissues for osmotic adjustments under stress. It thus contributes to enhanced abiotic stress tolerance [26,27]. *V. corymbosum* showed a wide opened stomatal gap with increasing heat stress, whereas *V. darrowii* showed a reduced gap (Figure 1B). In this research, the mean longitudinal distance between stomatal guard cells in *V. corymbosum* leaves increased along with the increased heat stress periods from 6- to 9-h, which indicates a shift in the pattern of stomata closure. In contrast, *V. darrowii* showed a reduced longitudinal distance with an increase in heat stress, which indicates a steady closing (Figure S1D). Heat stress-induced changes in stomata closure and low water loss rates have been reported in plants as a mechanism to overcome the impact of stress [28,29].

### 2.2. Differences in Gene Expression between V. corymbosum and V. darrowii at 6 and 9-h of Heat Stress

**Figure 2:**
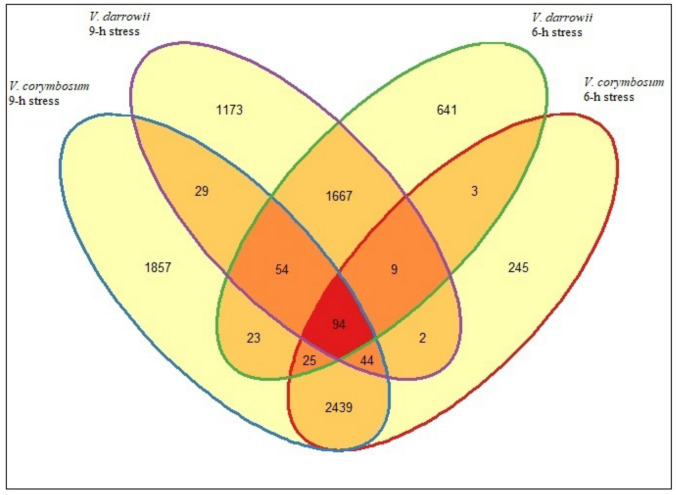
Venn diagram comparing the number of genes with statistically significant differential expression in response to heat stress at 6 and 9-h in *V. darrowii* and *V. corymbosum* species.

Transcriptome analysis yielded close to 65 million reads, with an average size of 1041 base pair contigs and 156,183 assembled transcripts in *V. corymbosum* and, approximately 70 million reads, with an average 1139 base pair contig length and 183,343 assembled transcripts in *V. darrowii* (Table 1). To identify the DEGs, the transcripts from the 6- and 9-h stress samples were compared against the control. *V. corymbosum* expressed 2861 DEGs at 6-h stress and 4565 DEGs at 9-h stress, with >3-fold differences in expression (*p* < 0.05). *V. darrowii* showed 2516 DEGs at 6-h stress and of 3072 DEGs at 9-h stress (Figure 2, Figure S2). Only 94 genes being commonly expressed between the two species indicated differential response mechanisms to heat stress. Uniquely expressed genes at 6-h stress were more numerous in *V. darrowii* than *V. corymbosum*. The top 10 and all the DEGs for both species are given in Table S1. The top DEGs in both species corresponded to heat shock protein (HSP) expression which is consistent with other abiotic stress studies [30]. HSP expression was evident in the heat stress response and recovery in grape leaves [30]. Thus, the identified DEGs highlight major changes in gene expression patterns in both genotypes and point to the differential response mechanisms adapted by the two blueberry species used in this experiment. Induced expression of HSPs in both the blueberry species suggesting the possible stomatal structure and regulation (opening and close) by HSP mediated protection under heat stress was evidenced (Figure 1, Figure S1C,D).

### 2.3. DEGs Commonly Enriched Pathways in Blueberry Species

**Figure 3:**
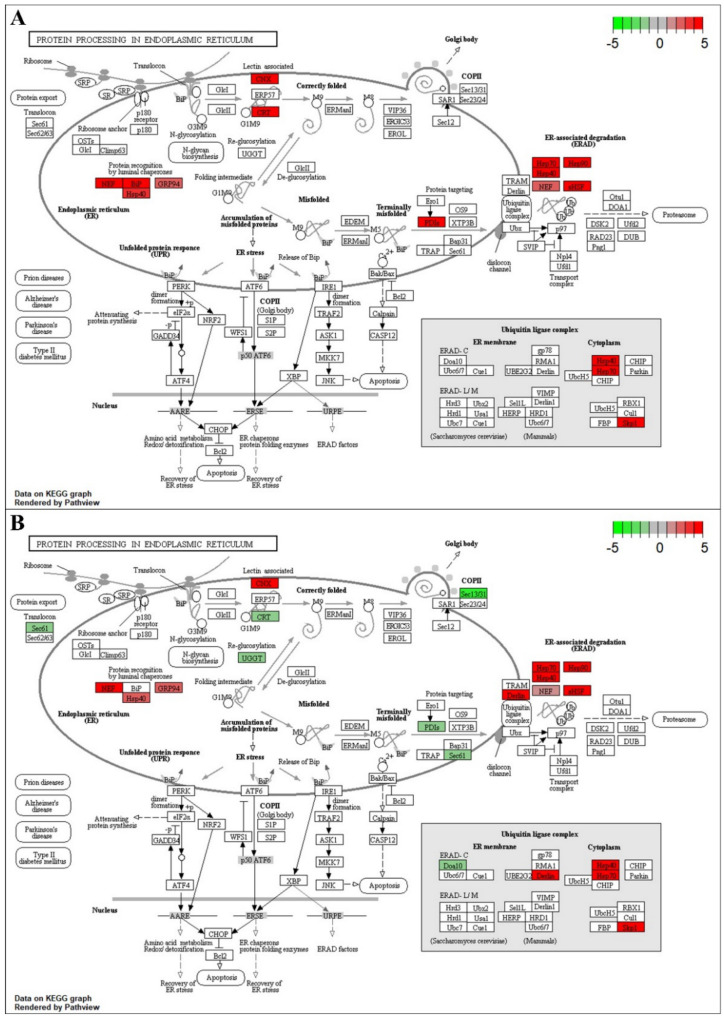
Protein processing in the endoplasmic reticulum pathway: (**A**) Map of alignment of *V. corymbosum* differentially expressed genes (DEGs) to *Vitis vinifera* genome at 6-h heat stress. (B) Map of alignment of *V. corymbosum* DEGs to *V. vinifera* genome at 9-h heat stress. The colored box represents DEGs, and their degree of expression is depicted by color variations, with red the most upregulated and green the most downregulated.

**Figure 4:**
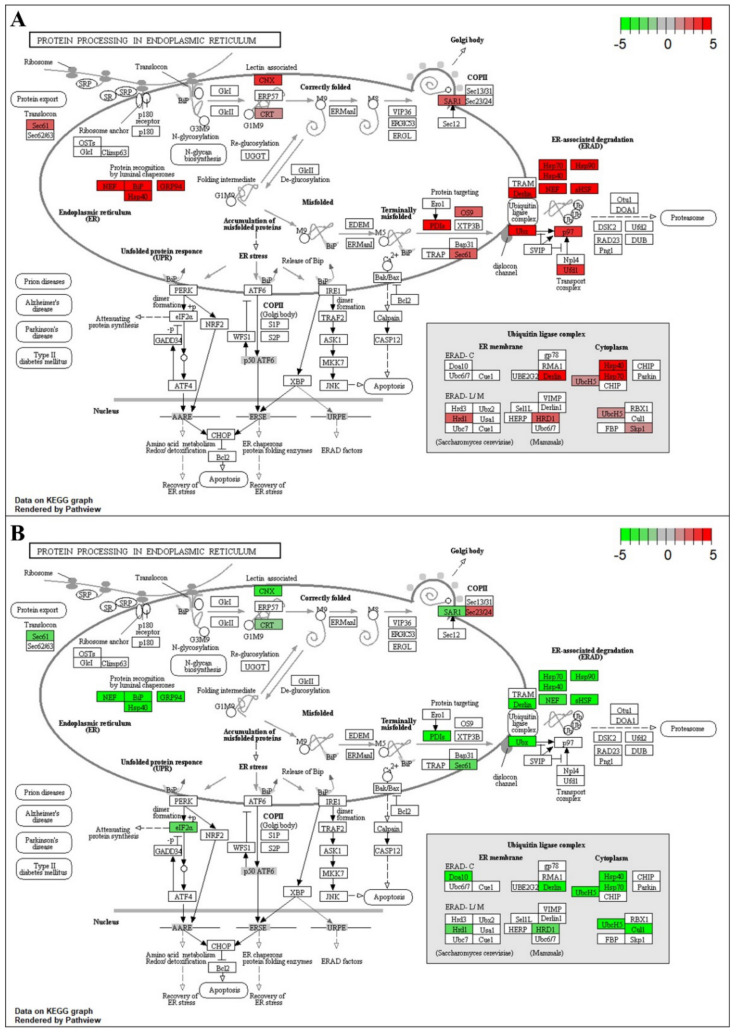
Protein processing in endoplasmic reticulum pathway: (**A**) Map of alignment of *V. darrowii* DEGs to *V. vinifera* genome at 6-h heat stress. (B) Map of alignment of *V. darrowii* DEGs to *V. vinifera* genome at 9-h heat stress. The colored box represents DEGs, and their degree of expression is depicted by color variations, with red the most upregulated (*p* < 0.05) and green the most downregulated (*p* < 0.05), white box represents no significant differences in gene expression.

Protein processing in the endoplasmic reticulum (ER) was a highly enriched pathway in both species (Figure 3 and Figure 4, Tables S2 and S3). Both blueberry species showed induced transcript abundance of genes encoding HSPs (Hsp40, Hsp70, and Hsp90), transcription factors (TFs), and small heat shock factors (sHSFs) at 6- and 9-h stress; however, the transcript levels of these genes showed a downregulation pattern in *V. darrowii* at 9-h stress. Moreover, both species showed upregulation of other genes involved in protein processing in the ER and the misfolded protein repair (*NEF*, *BiP*, *PDIs*, *GRP94*, *SKP1*, *CRT*, *CNX*) at 6-h stress, but *V. darrowii* showed a downregulation pattern of most of these genes except *GRP94* at 9-h stress. *V. darrowii* showed a unique upregulation of transcript patterns with protein processing in ER and ubiquitination genes (*Derlin, Sec 61, OS9, Ubx, p97, Ufd1, UbcH5, HRD1, SAR1*) at 6-h stress and downregulation at 9-h stress except for *Ubx* and *Ufd1* genes, which still maintained an upregulation pattern even at 9-h stress. Additionally, *Sec23/24* showed highly induced expression, whereas a ubiquitination gene (*cull*) and a unique TF, elongation factor (*eLF2*) showed a downregulation pattern at 9-h stress. Another distinctive ubiquitination gene, *Doa10*, showed a downregulation pattern at 9-h stress in both species.

In the ER, newly synthesized peptides are folded into proper functional proteins with the help of chaperone proteins. Chaperones play an important role in the repair of the misfolded proteins and homeostasis. In plants, HSPs/chaperones play an important role under heat stress and are responsible for the events of protein folding, assembly, translocation, and degradation [31,32]. We found an upregulation of sHSF/HSPs and molecular chaperones (*Hsp90*, *Hsp70*, *Hsp40*) with increased exposure to heat stress in both blueberry species (Figure 3 and Figure 4). Moreover, we observed the upregulation of other genes involved in protein processing in ER and misfolded protein repair in both the species at 6-h stress, but a downregulation pattern for most of these genes was observed at 9-h stress in *V. darrowii*. In contrast, other genes maintained upregulation patterns for protein processing in ER and ubiquitination (*Derlin*, *Sec 61*, *OS9*, *Ubx*, *p97*, *Ufd1*, *UbcH5*, *HRD1*, *SAR1*) in *V. darrowii* at 6-h stress, and a few specific genes showed upregulation (*Ubx*, *Ufd1*, and *Sec23/24*, *cull*) and downregulation (*eLF2*) patterns at 9-h stress. Previous studies reported DEGs involved in protein processing in ER such as protein homeostasis (sHSF), luminal chaperon (*NEF, GRP94*), chaperon binding protein (BiP), protein folding machinery (*CRT*, *CNX*, *PDIs*, *UGGT*, HSPs), protein translocation (*Sec* 61, 23/24), and ubiquitination (*Derlin*, *OS9*, *Ubx*, *p97, Ufd1*, *UbcH5*, *HRD1*, *SAR1*) and other mechanisms involved in the heat stress response in plants [33,34,35,36,37]. The differential expression patterns of protein processing genes in ER suggest a prevalence in differential response and possible protein homeostasis and repair mechanisms under heat stress in blueberry species.

### 2.4. Unique DEG-Enriched Pathways in V. corymbosum

The pathways for glycine, serine, and threonine metabolism, ascorbate, and aldarate metabolism, and fatty acid degradation were expressed at 6-h stress and the peroxisome pathway at 9-h stress in *V. corymbosum* (Tables S2 and S3). At 6-h stress, upregulated genes involved in the fatty acid degradation pathway were long-chain acyl-CoA synthetase (EC: 6.2.1.3), acetyl-CoA, acetoacetyl transferase (EC: 2.3.1.16), alcohol dehydrogenase (EC: 1.1.1.1), and aldehyde dehydrogenase (EC: 1.2.1.3) (Figure S3). Several transcriptomic studies reported DEGs in the fatty acid degradation pathway and significant upregulation of multiple genes and their possible signatures in response to heat stress in plants [38,39,40]. Heat stress causes loss of membrane integrity and stability, contributing to decreased plant physiological processes. The membrane stability and integrity are determined by the internal chemical composition (phospholipids or fatty acids) [40]. Hence, maintaining fatty acid synthesis and the fate of the degradation is an important biological process under heat stress. In our study, fatty acid degradation was significantly affected during heat stress at 6-h in *V. corymbosum*. Four unigenes belonging to 3 categories of fatty acid metabolism showed altered expression during heat stress (Figure S3). These include genes involved in fatty acid biosynthesis (long-chain acyl-CoA synthetase), fatty acid elongation (acetyl-CoA, acetoacetyl transferase), and fatty acid degradation (alcohol dehydrogenase and aldehyde dehydrogenase), which suggests the homeostasis and saturation of fatty acids and their role in thermotolerance.

The glycine, serine, and threonine metabolism pathway was the second most expressed pathway in *V. corymbosum* at 6-h stress. In this pathway, eight genes encoding the specific enzymes were significantly upregulated, and one gene was downregulated at 6-h stress (Figure S4). We found upregulation of genes involved in the production of serine, tryptophan, alanine, and glyoxylate [hydroxypyruvate reductase (EC: 1.1.1.81), tryptophan synthase beta chain (EC: 4.2.1.20) and glyoxylate aminotransferase (EC: 2.6.1.44)], and threonine and glycine [(threonine aldolase (EC: 4.1.2.48), homoserine kinase (EC: 2.7.1.39)], valine, leucine, and isoleucine biosynthesis and metabolism of propanoate, cysteine, and methionine [threonine dehydratase (EC: 4.3.1.19)]. We also found upregulation of the gene that encodes the precursor molecule (3P-D-glycerate) for several amino acid biosynthesis genes [phosphoglycerate mutase (EC: 5.4.2.12)] and genes encoding biosynthesis of aspartate amino acids (lycine and methionine, threonine) [aspartate kinase (EC: 2.7.2.4)]. Genes involved in glycine biosynthesis and signaling [sarcosine oxidase/L-pipecolate oxidase (EC: 1.5.3.1)] were downregulated. The pathways of glycine, serine, and threonine metabolism were significantly enriched in *V. corymbosum* at 6-h stress and included several genes involved in glyoxylate and dicarboxylate metabolism (hydroxypyruvate reductase), pyruvate/glycolysis (phosphoglycerate mutase); valine, leucine, and isoleucine biosynthesis, and cysteine and methionine metabolism (serine/threonine dehydratase); alanine, aspartate, and glutamate metabolism (alanine/glyoxylate aminotransferase); glycine/threonine biosynthesis (homoserine kinase, threonine aldolase); and lysine biosynthesis (aspartate kinase). Similarly, DEGs enriched in glycine, serine, and threonine metabolism pathways were previously reported in plants in response to heat stress [41,42].

Increased expression of genes involved in glycine, serine, and threonine metabolism under heat stress suggests the possible role of these enzyme activities and their influence on promoting some essential amino acid transportation and synthesis in response to heat stress. Previous studies in plants reported a considerable effect and possible mechanisms with the induction of amino acid synthesis in response to heat stress in plants [43,44]. We found an upregulation pattern of genes encoding enzymes involved in ascorbate and aldarate metabolism, such as UDP-sugar pyrophosphorylase (EC: 2.7.7.64), L-ascorbate peroxidase (EC: 1.11.1.11), and aldehyde dehydrogenase (NAD+) (EC: 1.2.1.3), and downregulation of GDP-L-galactose phosphorylase (EC: 2.7.7.69) at 6-h stress (Figure S5). The enriched pathway of ascorbate and aldarate metabolism in *V. corymbosum* at 6-h stress, the enhanced expression of genes involved in ascorbate/carbohydrate metabolism (L-ascorbate peroxidase), several plant metabolite synthesis genes (aldehyde dehydrogenase), the genes involved in sugar metabolism (UDP-sugar pyrophosphorylase) and the downregulation of ascorbate biosynthesis gene (GDP-L-galactose phosphorylase (EC: 2.7.7.69) suggest compensation mechanisms of ascorbate synthesis under heat stress. Induction of ascorbate metabolism genes and ascorbate as a potential antioxidant for scavenging reactive oxygen species under heat stress have been well studied in plants [45,46].

The peroxisome pathway was a key enriched pathway in *V. corymbosum* at 9-h stress; it plays an important role in the oxidation of cellular components such as fatty acids, lipids, and amino acids to produce alternate components [47,48]. We found an upregulation pattern of a matrix protein importer (peroxin; PEX5, PEX7), membrane protein importer (PEX5), enzymes involved in fatty acid oxidation [acetyl-CoA acyltransferase 1 (ACAA1), peroxisomal 2,4-dienoyl-CoA reductase (PDCR), long-chain acyl-CoA synthetase (ACLS)], and sterol precursor biosynthesis genes [mevalonate kinase (*MVK*)]. We also found downregulation of amino acid metabolism genes [L-pipecolate oxidase gene (*PIPOX*)] and antioxidant system genes [superoxide dismutase (SOD)] (Figure S6). The key enriched DEGs (eight genes) were in peroxisome metabolism pathways in *V. corymbosum* at 9-h stress (Figure S6). Peroxisomes are highly metabolic organelles surrounded by a single membrane and play key roles in metabolism because they are involved in many processes, including fatty acid β-oxidation (ACAA1, ACLS, PDCR), glyoxylate cycles, photorespiration, polyamine metabolism, and biosynthesis of phytohormones (MVK). They also act as a site for antioxidant enzymes (SOD) [47,48]. We found upregulation of several genes encoding enzymes involved in various functions in peroxisomes such as matrix protein transport receptors (PEX5, PEX7), membrane protein transporter (PEX5), fatty acid β-oxidation (ACAA1, ACLS, PDCR), and biosynthesis of phytohormones (MVK) and downregulation of antioxidant enzyme (SOD) and amino acid metabolism (PIPOX) genes in *V. corymbosum* at 9-h stress. These findings suggest that stress signal response, fatty acid metabolism, hormone synthesis, and antioxidant machinery were the mechanisms induced in response to heat stress. Previous findings also illustrated the top enriched peroxisome pathway and DEGs in response to heat stress [38]. These results in *V. corymbosum* were evidenced by regulation of stomatal opening by the upregulation of genes involved in effective stress signaling, hormone synthesis (glycine, serine, and threonine metabolism, ascorbate, and aldarate metabolism pathway genes), and internal organelle structure stability possibly by the upregulation of fatty acid and peroxisome metabolism pathway genes (Figure 1 and Figure S1).

### 2.5. DEG Enriched Pathways in V. darrowii

The ribosome pathway was enriched at 6-h stress, but the photosynthesis-antenna protein and circadian rhythm-plant pathways were enriched at 9-h stress in *V. darrowii*. The ribosome pathway was the most cohesive pathway at 6-h stress (Figure S7, Tables S2 and S3) and showed a differential expression of genes that encode various ribosomal subunit proteins (small and large). The responsive genes encoding large ribosomal subunits (i.e., L3e, L4e, L8e, L5e, L9e, L10, L11, L13Ae, L15, L17e, L18Ae, L21e, L22e, L24, L26e, L27e, L32e, L34e, L40e, LF1, and LF2) were upregulated, and L14 was downregulated. Apart from the larger subunit responsive genes, the DEGs of smaller subunits (i.e., S5e, S6e, S7e, S9, S16e, S19e, S24e, S25e, S27e, and S27Ae) were upregulated, and S4, S12e, and S17 were downregulated. Previous studies also demonstrated that the ribosome pathway was enriched in response to heat stress [49]. We found 21 transcripts of large and 10 transcripts of small subunits corresponding to ribosomal proteins showing an upregulated expression pattern, and one large and three small subunit genes were showing downregulation in *V. darrowii* at 6-h stress as an early response. This finding suggests translational regulation as a response to heat stress mediated by ribosomal proteins. In Arabidopsis, ribosomal proteins are induced in response to heat stress, which suggests changes in expression of ribosomal proteins and subunits related to altered composition of the ribosomal subunits, and therefore translational regulation [50,51,52].

DEGs were enriched in the photosynthesis-antenna protein pathway in *V. darrowii* at 9-h stress. Predominantly, light-harvesting chlorophyll (LHC) a/b binding proteins were upregulated in the photosynthesis-antenna protein pathway. In total, seven genes were highly upregulated among them, three belonging to LHCa binding protein (Lhca3, Lhca4, and Lhca5) and four belonging to LHCb binding protein (Lhcb1, Lhcb2, Lhcb3, and Lhcb5) (Figure S8). Several other studies confirm enriched photosynthesis-antenna protein pathway and upregulation of LHCs in plants in response to heat stress [49,53].

In plants, PSII is the most sensitive component to high temperatures in the photosynthetic apparatus [54]. It plays an essential role in photosynthesis under heat stress [55]. LHCa/b binding proteins, which are key components of light-harvesting antennae of PSII, play various roles in regulating light-harvesting events, such as optimizing light energy utilization and dissipation of excessive light [56]. Heat stress/high intense light-induced ROS interact with proteins and lipids, thus inducing photo-oxidation and damaging chloroplasts. Hence, maintaining the structural and functional properties of LHC protein complexes is a prerequisite for avoiding photodamage caused by alleviating excitation energy pressure under heat stress [57]. In the present study, the upregulation of LHCa/b binding protein-encoding genes indicates overcoming heat stress-induced photo-oxidation in *V. darrowii*, which might be the most fundamental pathway involved in heat tolerance. In this context, prolonged heat stress in *V. darrowii* may cause the differential expression of ribosomal proteins, and translational regulation and maintenance of the LHC protein complex, which may be important events responding to heat stress. The DEGs in the photosynthesis-antenna protein pathway responsible for light harvest and photosynthesis (PSII) possibly protect the chloroplast membrane structures in *V. darrowii* (Figure 1).

Four genes (*CRY*, *CK2α*, *HY5*, and *CHS*) from the circadian-rhythm plant pathway were found upregulated during 9-h stress in *V. darrowii* (Figure S9). These genes included encoding pigments, cryptochromes (CRY), enzymes chalcone synthase (CHS) and casein kinase II subunit alpha (CK2α), and the transcription factor ELONGATED HYPOCOTYL5 (HY5). Several studies reported that heat stress-induced DEGs were enriched in the circadian-rhythm plant pathway and contributed to time of day and night in circadian clock regulation and transcriptomic changes in response to heat stress in plants [58,59]. As a photoreceptor, CRY regulates light responses, including circadian rhythm, tropical growth, stomata opening, guard cell development, and abiotic stress responses. The photoexcited CRY network with signaling partner proteins alters gene expression at both transcriptional and translational levels and thus regulates the confirming metabolic and developmental programs in plants to respond to thermal adaptation with a plastic circadian clock [60]. Higher expression of CRYs in the *V. darrowii*, possibly regulating the stomata function under heat stress, as evidenced by the LSCM images showing the stomata closure with the increasing temperature (Figure 1B). CHS expression acts as a rate-limiting step in flavonoid biosynthesis, which is a major photo protectant in plants, and differential expression of CHSs mediates circadian rhythms under heat stress [61,62,63]. CK2α triggers temperature compensation of circadian rhythms via abscisic acid signaling in plants [64,65]. The transcription factor HY5 integrates the functions of different photoreceptors and controls the circadian rhythm-gated reactions that synergize responses between heat shock and light [66]. Induced expression of circadian-rhythm pathway genes suggests the regulation of photoreceptors and abscisic acid signaling and heat shock and light-induced responses with possibly a late responsive mechanism under heat stress.

### 2.6. Dynamics of Key Pathways Involved in Response to Heat Stress

Transcripts with corresponding ECs were searched in the KEGG enzyme database to explore related metabolic and biosynthesis pathways. In *V. corymbosum*, we found 928 DEGs corresponding to ECs of 42 categories of metabolic pathways and 10 categories of biosynthesis pathways. In *V. darrowii*, 1243 DEGs corresponded with ECs of >70 distinct metabolic categories and 27 biosynthesis pathways (Table S4, Figures S10 and S11).

**Figure 5:**
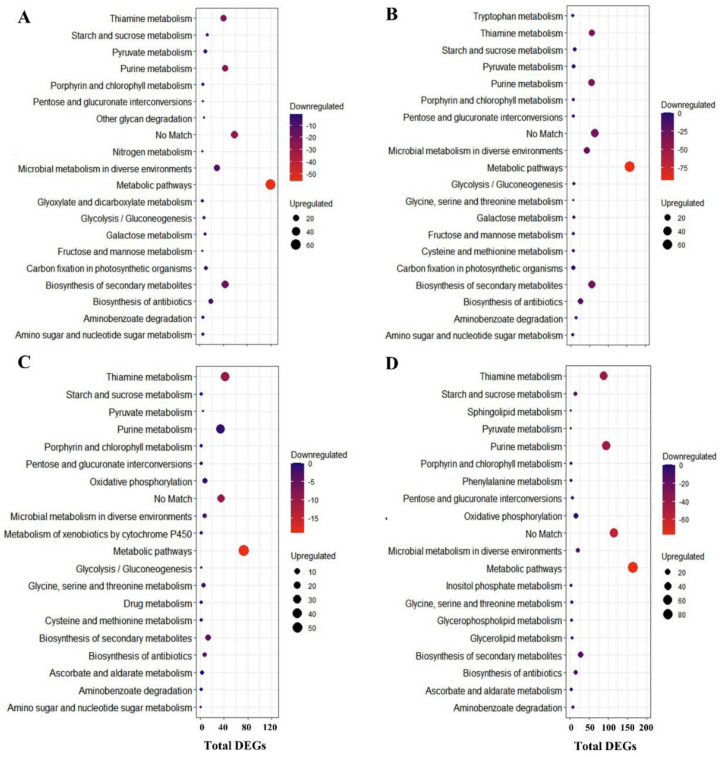
DEGs enriched in the top 20 metabolic pathways. Highly enriched pathways among the down- and upregulated DEGs represented based on the counts. Counts indicate the number of DEGs annotated in each pathway, and the circle colour blue indicates minimal downregulated gene count and red color shows the maximum number of downregulated gene count. The circle size depicts upregulated gene counts in each pathway, and the position of the circle depicts the total number of DEGs. (**A**) DEGs in metabolic pathways of *V. corymbosum* at 6-h heat stress, (**B**) DEGs in metabolic pathways of *V. corymbosum* at 9-h heat stress, (**C**) DEGs in metabolic pathways of *V. darrowii* at 6-h heat stress, (**D**) DEGs in metabolic pathways of *V. darrowii* at 9-h heat stress.

*V. corymbosum* expressed 92 active metabolic pathways during heat stress, whereas *V. darrowii* expressed 137 metabolic pathways (Table S4). Among top 20 pathways at both 6- and 9-h stress, 12 were common between the species and are mainly related to purine and thiamine metabolism pathways, biosynthesis of secondary metabolites, biosynthesis of antibiotics, starch and sucrose metabolism, aminobenzoate degradation, pentose and glucuronate interconversions, and porphyrin and chlorophyll metabolism (Figure 5, Table S4). Previous studies also reported that these metabolic pathways correspond to heat stress in plants as the top representative pathways [67,68,69]. Oxidative phosphorylation, ascorbate, and aldarate metabolism, glycerolipid and glycerophospholipid metabolism, inositol phosphate metabolism, metabolism of xenobiotics by cytochrome P450, and phenylalanine metabolism were unique to *V. corymbosum* (Figure 5A,B). The DEGs involved in oxidative phosphorylation, lipid, sugar, amino acid, and secondary metabolite pathways were possibly maintaining the osmotic potential in the stomatal cells that was evidenced by the lack of changes in stomata, and their organelle membrane structures captured using LSCM images in *V. corymbosum* (Figure 1).

The enzymes involved in oxidative phosphorylation play an essential role in oxidative respiration under heat stress [70]. In plants, inositol phosphate and phenylalanine-induced transcripts and their osmoregulation mechanisms were reported in response to heat stress [70,71,72]. Extensively studied genes in plants include induced expression of cytochrome P450 genes involved in the biosynthesis of indole alkaloid as well as other secondary metabolites and glycerolipid- and glycerophospholipid-induced transcripts in lipid metabolism under heat stress and their mechanisms in response to heat stress [73,74,75]. The unique metabolic pathways in *V. darrowii* are carbon fixation, pyruvate, amino sugar, nucleotide sugar, galactose, glyoxylate and dicarboxylate, fructose and mannose metabolism, other glycan degradation, and nitrogen metabolism (Figure 5C,D). Similarly, previous studies reported that DEGs of the carbon fixation pathway respond to heat stress in plants and have possible regulation mechanisms [41,76,77]. Photosynthesis enhancement by ameliorating glyoxylate and dicarboxylate metabolism genes and their induced transcripts are involved in the comprehensive regulation of photosynthetic carbon metabolism under heat stress [37,70,77]. Previous studies reported fructose and mannose metabolism gene expression in response to heat stress and their osmoregulation [74,78].

The number of biosynthesis pathways with DEGs were higher in *V. darrowii* than *V. corymbosum* (22 vs. 15); 12 biosynthetic pathways were common to both species (Table S4). The biosynthesis pathways common to both the species were phenylpropanoid, ubiquinone, terpenoid, terpenoid-quinone, sesquiterpenoid- and triterpenoid-related, isoquinoline and alkaloid, amino acid (lysine, phenylalanine, tyrosine, and tryptophan), wax synthesis (cutin and suberin), and hormone (steroid) synthesis. In particular, *V. darrowii* featured fatty acid, flavonoid, anthocyanin biosynthesis, carotenoid, folate, glucosinolate, indole alkaloids, diterpenoid, valine, leucine, and isoleucine biosynthesis (Figures S10 and S11). Biosynthetic pathways [amino acid (lysine, phenylalanine, tyrosine, and tryptophan), wax (cutin and suberin), and hormone (steroid)] have been reported as the most stress-responsive under heat stress in plants [69,72,79].

The specific biosynthesis pathways in *V. darrowii* consisted of fructose and mannose metabolism; other glycan degradation; fatty acid, flavonoid, and anthocyanin biosynthesis; carotenoid, folate, and glucosinolate indole alkaloids; and diterpenoid, valine, leucine, and isoleucine biosynthesis (Figures S10 and S11). Similar findings of DEGs in fatty acid biosynthesis and their important role in lipid membrane protein stability and wax biosynthesis in response to heat stress have been reported [75,79,80,81]. The wax synthesis that was evidenced by the glassy wax coating on the *V. darrowii* leaves was probably due to high number of DEGs involved in wax and fatty acid biosynthesis pathway. DEGs in several non-enzymatic antioxidant biosynthesis pathways (flavonoid, anthocyanin, carotenoid, and other secondary metabolites) and their essential role in the scavenging of ROS to overcome the heat stress impacts is evidenced by the accumulation of antioxidant compounds under heat stress in plants [81,82]. DEGs were identified in the valine, leucine, and isoleucine biosynthesis pathways and their induction in response to osmoregulation under heat stress [83]. Several studies reported the differences between genotypes for differentially expressed transcripts in response to heat stress and other abiotic stresses as well as maintenance of different mechanisms to cope with the stress impacts [36,44,84,85]. These results suggest that the differential expression levels of genes involved in different biological pathways can help in understanding the variation in response to heat stress in the blueberry species. The transcriptomic data also provided the basis for investigating gene-level changes and regulation mechanisms of specific metabolic and biosynthetic pathways in response to heat stress.

### 2.7. GO Enrichment Analysis of Heat Responsive DEGs

**Figure 6:**
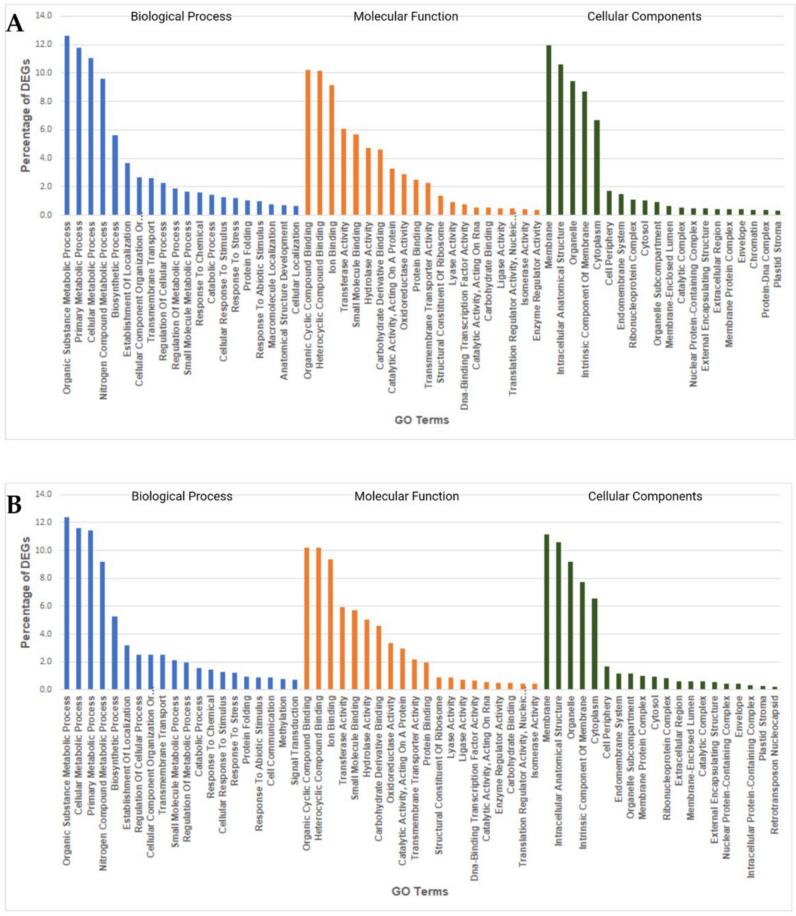
Gene ontology enrichment analysis of DEGs from *V. corymbosum***.** Highly enriched Gene Ontology (GO) terms among the DEGs from *V. corymbosum* under 6-h heat stress (**A**) and 9-h heat stress (**B**).

**Figure 7:**
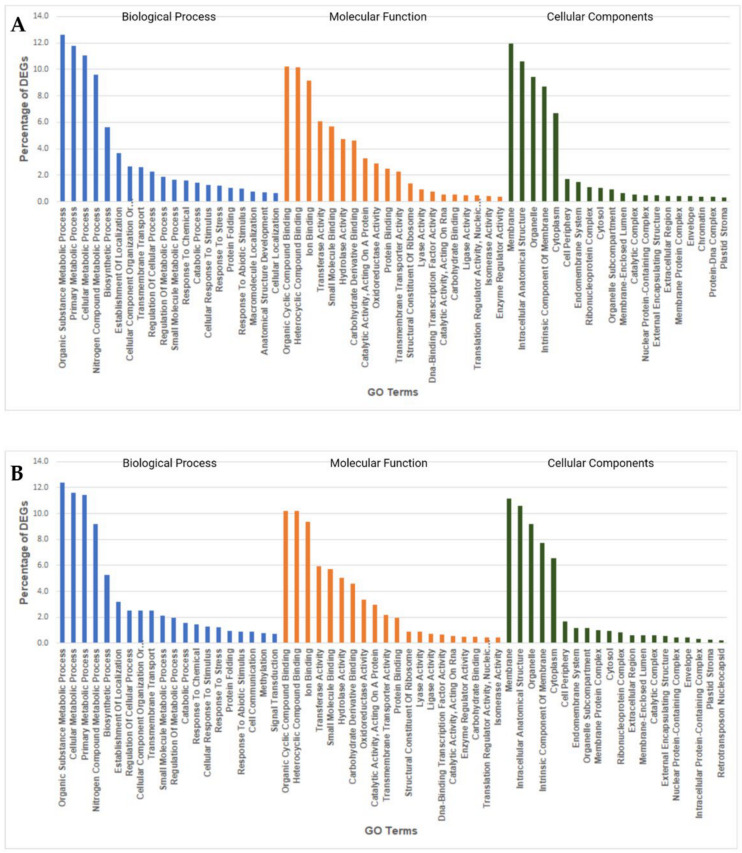
Gene ontology enrichment analysis of DEGs from *V. darrowii***.** Highly enriched Gene Ontology (GO) terms among the DEGs from *V. darrowii* under 6-h heat stress (**A**) and 9-h heat stress (**B**).

Gene Ontology (GO) enrichment analysis helps to understand DEGs regulation under the three essential categories: biological processes, molecular functions, and cellular components. GO enrichment analysis of heat-responsive DEGs from both species revealed the functional categories activated with heat stress under 6-h and 9-h stress treatments. The significantly enriched biological processes in *V. corymbosum* upon heat stress included several stress response processes such as organic substance metabolic process (7.7%), nitrogen compound metabolic process (6.1%), response to stress (0.9%), and response to an abiotic stimulus (0.5%). Organic cyclic compound binding (7.7%) and heterocyclic compound binding (7.7%) contained more DEGs under molecular functions in *V. corymbosum*. Cellular components category enriched with DEGs for membrane (8.9%), intracellular anatomical structure (6.2%), an intrinsic component of membrane (5.9%), and organelle (5.5%) (Figure 6). The GO terms enriched in *V. darrowii* under heat stress were similar to *V. corymbosum* but contained more DEGs under the many enriched GO terms than *V. corymbosum*. Biological processes in *V. darrowii* included organic substance metabolic process (12.6%), nitrogen compound metabolic process (9.6%), response to stress (1.2%), and response to an abiotic stimulus (0.9%). Membrane (11%) associated DEGs were higher in *V. darrowii*, followed by intracellular anatomical structure (10.5%) (Figure 7). These results suggested that high temperature treatment induced changes in biological, molecular, and cellular processes that were common and unique between the species. Furthermore, under heat stress, the genes involved in the membrane associated DEGs were probably activated to alleviate the stress damage in both species. In agreement with the LSCM image analysis of stomata organelle structural modifications in *V. darrowii* and the presence of a high percentage DEGs in intra-cellular anatomical structure under heat stress suggest s the possible role in thermal adoptation (Figure 1). In plants, the previous studies reported that there were similar GO enriched analysis and expression of DEGs in biological, molecular, and cellular processess under heat stress [68,69].

### 2.8. Quantitative Real-Time PCR (RT-qPCR) Validation of DEGs from RNAseq

**Figure 8:**
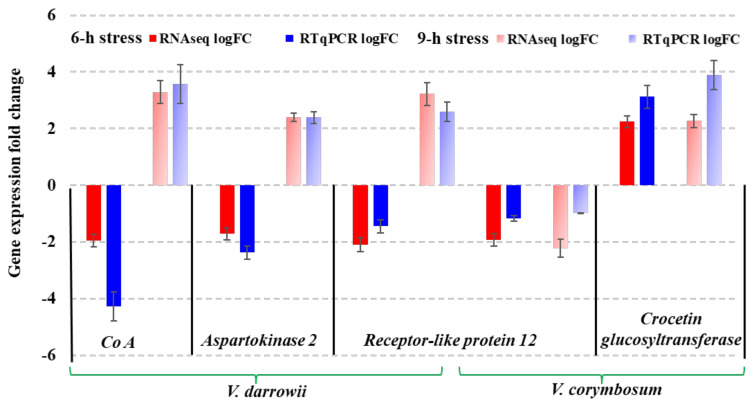
RT-qPCR validation of DEGs: Gene expression patterns of randomly selected genes in both blueberry species (*V. darrowii* and *V. corymbosum*) at 6- and 9-h heat stress.

Four DEGs were randomly selected for RT-qPCR analysis to validate gene expression patterns revealed by RNAseq analysis. The genes receptor-like protein 12, CoA (EC: 6.2.1.12), and aspartokinase 2 (EC: 2.7.2.4) were used to validate the expression profiles in *V. darrowii* and receptor-like protein 12 and crocetin glucosyltransferase in *V. corymbosum* for a total of 20 events. The blueberry actin was used as the housekeeping gene control. The gene expression patterns from RT-qPCR analysis of the 4 randomly selected genes were consistent with the DEG profiles of RNAseq results (Figure 8). The expression of these genes reveals their role in specific pathways in response to heat stress in the blueberry species.

## 3. Materials and Methods

### 3.1. Plant Materials and Phenotypic Characterization

Multiple *V. corymbosum* and *V. darrowii* plants were generated from softwood cuttings and kept on a mist bench for 6 months at the Philip E. Marucci Center for Blueberry and Cranberry Research and Extension at Rutgers University, Chatsworth, NJ, USA (39.71543° N, 74.51046 ° W). Three months before experimentation, plants were brought to the Department of Agriculture and Natural Resources at Delaware State University, Dover, DE, USA (39.1861° N, 75.5423° W), and maintained outside the greenhouse under natural conditions. The plants selected for the experiment were also characterized by their morphological traits such as leaf color, size, plant height, and stem color.

### 3.2. Heat Stress Induction

Prior to heat stress evaluation, 8-month-old plants were maintained under controlled-environment chambers (Conviron Model PGR15; Winnipeg, MB, Canada) adjusted to ambient room temperature of 25 °C for 1.5 h, and control samples (0 h) were collected for all experiments. Then sets of plants were exposed to heat stress at 45 °C for 6 and 9 h, with 70% humidity and light intensity 3000 µmol m^−2^ s^−1^. The leaf samples were collected at both 6 and 9 h for physiological and molecular studies.

### 3.3. Stomata and Internal Organelle Image Assay

A random number generator was used to select flash-frozen leaves within a gridded mortar. Five leaves were selected from each biological replication, and 10 images for stomata opening and 10 z-stacks of internal stomata organelle structures. Leaves were placed with stomata facing down in a chambered coverglass (Nunc, Lab-Tek, Thermo Scientific, Waltham, MA, USA). A drop of water was placed on the leaf sample, and a glass block was placed on top of the sample to hold close to the coverslip. Images of auto-fluorescing chloroplasts were obtained for each leaf by using a ZEISS LSM 510 laser scanning confocal microscope (Carl Zeiss Micro-imaging GmbH, Jena, Germany). Samples were imaged with a Zeiss C-Apochromat 40X/1.2 N.A (Carl Zeiss Micro-imaging GmbH, Jena, Germany). Water objective, with a zoom factor of 2. To facilitate image rendering and Z-stack imaging, we used the Zen 2009 software package (Zeiss, Jena, Germany). Final image stacks were deconvolved by using Huygens 19.04 (Scientific Volume Imaging, Hilversum, Netherlands,). Volume rendering and surface area calculations involved using Imaris 9.5 (Oxford Instruments, Bitplane AG, Zurich, Switzerland). Stomata opening was visualized by using differential interference contrast (DIC) images, and the opening (longitudinal distance) was measured by using Zen 2009. Samples were imaged and analyzed at the Optical Science Center for Applied Research at Delaware State University, Dover, DE, USA.

### 3.4. RNA Isolation, Library Preparation, and Illumina Sequencing

Total RNA was extracted from leaf material by the modified CTAB method [86], treated with DNAse I, and purified with RNeasy MinElute Cleanup Kit (Qiagen, Germantown, MD, USA). Two biological replicates were used for each of the control and treatment conditions. The quantity of RNA was assessed by measuring the optical density (OD) with a Nanodrop-2000 Ultraviolet Spectrophotometer (Thermo Fisher, Waltham, MA, USA). The purity and integrity of total RNA were measured by agarose gel electrophoresis and the Agilent 2100 Bioanalyzer. RNA integrity number (RIN) values were calculated by Eukaryote Total RNA Nano Assay (Agilent Technologies, Santa Clara, CA, USA). The NEBNext Ultra II RNA Library Prep Kit was used to prepare RNA sequencing libraries for each sample according to the manufacturer’s specifications (New England BioLabs, Ipswich, MA, USA). Isolation and fragmentation of mRNA from total RNA involved using magnetic beads with Oligo and fragmentation buffer. Random hexamer primers were used to synthesize cDNA from fragmented mRNA, and double-stranded cDNA was end-repaired and ligated with Illumina sequencing adaptors. Libraries were amplified with sequencing primers, and library quality was analyzed by using the Bioanalyzer. The Invitrogen Qubit 4 Fluorometer was used for library quantification, and libraries were sequenced by using the Illumina NextSeq 500 platform according to the manufacturer’s specifications for paired-end sequencing. The FASTQ files were generated with the Illumina bcl2fastq tool, and a minimum of 25 million paired-end reads were generated for each of the samples. Cutadapt was used to remove adaptors, and “sickle” was used to remove low-quality reads (Phred score QV < 30) [87,88].

### 3.5. De novo Assembly and Clustering

The Trinity de novo assembler and CD-HIT-EST were used for the de novo assembly and clustering of the transcripts using default parameters as described in the software manual [89,90,91,92,93,94,95]. DEGs were identified from pair-wise comparison of control and treatment conditions using the edgeR package [96]. The raw counts were normalized using Trimmed Mean of M-values (TMM) and filtered using count per million (CPM) of 1. DEGs were selected with a cut-off value of log fold-change (FC) > 1 and false discovery rate (FDR) < 0.05. Annotation for experimental transcripts involved using the National Centre for Biotechnology Information (NCBI) BLASTX tool from the BLAST+ package, which identified significant matches to transcriptomes in our dataset. BLAST2GO was used to generate annotation files; all transcripts expressed in each species were listed in annotation documents. Annotation information from the BLAST2GO suit included unique enzyme codes (ECs) that are compatible with the Kyoto Encyclopedia of Genes and Genomics (KEGG). DEGs were aligned to the *Vitis vinifera* and *Arabidopsis thaliana* genomes for comprehensive analysis.

### 3.6. Pathway Analysis

For metabolism and biosynthesis pathway analysis, identified DEGs were matched to annotation files, and corresponding ECs were used in the KEGG database to identify metabolic and biosynthesis pathways associated with corresponding transcripts. Pathway correspondence in each time period was counted, measuring overall activity. For pathway enrichment analysis, DEGs aligned to the *V. vinifera,* and *A. thaliana* genome were matched to the gene IDs given in the NCBI Entrez database. The R packages clusterProfiler and pathview were used to cluster and generate pathway maps [97,98]. Pathway enrichment analysis using clusterProfiler relies on K means clustering with FDR and logFC as controls. Pathway IDs produced from enrichment analysis were used to develop pathway enrichment maps with path view by using NCBI gene IDs and *p*-values as controls [98,99,100].

### 3.7. Real-Time Quantitative PCR (RT-qPCR)

RT-qPCR was performed to validate the genes identified by using transcriptome sequencing analysis. Total RNA from three biological replicates of leaf tissue per treatment and the control sample was extracted by using the RNeasy Plant Mini Kit (Qiagen, Germantown, MD, USA). Total RNA was treated with DNAseI (Qiagen, Germantown, MD, USA) and purified and analyzed with RNeasy MiniElute Cleanup Kit (Qiagen, Germantown, MD, USA). cDNA was synthesized by using RevertAid First Strand cDNA Synthesis Kit according to the manufacturer’s guidelines (Thermo Scientific, Waltham, MA, USA). The primers were designed from assembled sequences by using Primer 3 software and synthesized from IDT (Integrated DNA Technologies, Coralville, IA, USA). The primers for *Actin* (a housekeeping gene) and five candidate genes from RNAseq analysis are listed inTable S5. Quantitative gene expression was analyzed with a RT-qPCR system (Thermo Fisher Scientific, Applied Biosystems, Waltham, MA, USA). Three technical replicates were used for each biological replicates, and RT-qPCR was performed with 8 μL of a w/ROX master mixture with the SuperScript III Platinum SYBR Green One-Step qPCR Kit (Invitrogen, Thermo Fisher Scientific, Waltham, MA, USA) containing 2 μL each forward and reverse primers (5 μM) and 20 ng cDNA (2 μL) to make the final reaction volume of 14 μL. RT-qPCR (ThermoFisher Scientific, Applied Biosystems, USA) was performed at 95 °C for 5 min, and 40 cycles of 95 °C for 30 s and 58 °C for 30 s. Relative gene expression was quantified by the 2^−ΔΔCt^ method, and FC in expression was compared to the expression of control samples (0 h) [101]. The selected candidate gene expression was normalized with *Actin* as an internal reference gene.

## 4. Conclusions

**Figure 9:**
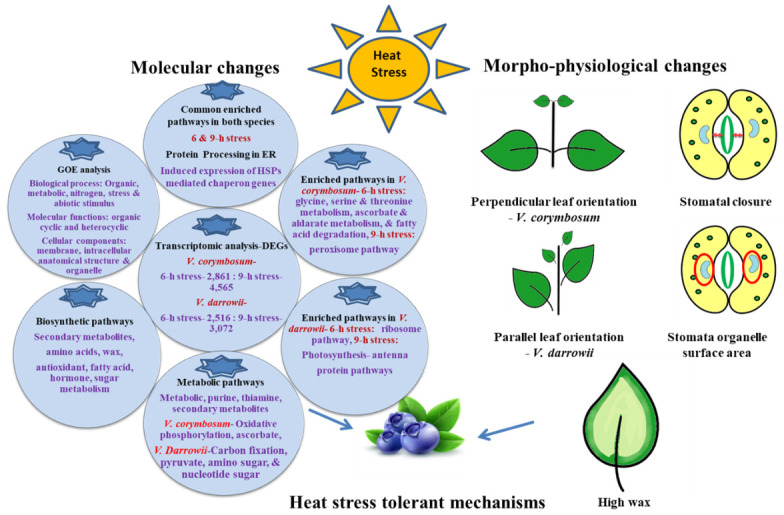
Schematic representation of differential morpho-physiological and molecular mechanisms involved in response to heat stress in the blueberry species.

This is the first transcriptome level study in blueberries against heat stress. We tried to provide comprehensive insights into the morpho-physiological and transcriptomic mechanisms underlying the heat stress response in two blueberry species. The differential responses to heat stress in blueberry species suggested improved thermal adaptation signatures in *V. darrowii* versus *V. corymbosum*. The evolved morpho-physiological adaptations of small leaf size, clustered parallel leaf orientation, waxy layer, increased surface area, and stomatal closing in *V. darrowii* possibly protects against heat stress. The transcriptome profiling revealed a varied response in terms of DEGs in *V. darrowii* and *V. corymbosum.* The highly enriched DEGs were in protein processing in the ER pathway regardless of species or sampling times, which suggests the prevalence of possible HSP-mediated protein homeostasis and repair mechanisms under heat stress. As evidenced in the differential expression of several metabolic pathways in these contrasting species (serine, threonine, ascorbate and aldarate metabolism, and fatty acid degradation in *V. corymbosum* and DEGs in ribosome pathways, the expression of photosynthesis-antenna proteins and circadian pathways in *V. darrowii* alone) the presence of variation in response to heat stress. A high number of active metabolic and biosynthesis pathways were enriched in *V. darrowii* versus *V. corymbosum*. The significant GO terms enriched DEGs were observed in biological processes such as organic, nitrogen compound metabolic process, stress stimuli and molecular functions such as organic cyclic and heterocyclic compound binding and cellular components such as membrane, intracellular anatomical structure, and organelle. The overall responses of blueberries to heat stress were summarized in Figure 9. This study reveals several differentially expressed genes and pathways, both temporal and species wise during heat stress. We hope this study will lay a foundation for further understanding of the heat stress tolerance in blueberries as they require relatively low temperatures for flower bud initiation and are exposed to higher temperatures in summers during fruit development stage.

## Figures and Tables

**Table 1:** Summary of transcriptomes of *V. corymbosum* and *V. darrowii* at 0, 6 and 9-h of heat stress.

Organism	*V. corymbosum*	*V. darrowii*
Total number of reads	64,979,396	70,331,700
Total base pairs (bp)	9,626,737,814	10,494,423,421
Total number of assembled transcripts	156,183	183,343
Total assembled bases	162,599,434	208,966,135
Average contig length (bp)	1041	1139
Total number of assembled genes	99,093	109,193
Percent GC (%)	41.6	41.6
Contig N50 (bp)	1768	1967

## Data Availability

The raw paired-end Illumina RNA sequencing reads generated in the current study are available in the Sequence Read Archive (SRA) at NCBI under the Bio project accession nos. PRJNA691351 (*Vaccinium corymbosum*) and PRJNA691352 (*Vaccinium darrowii*).

## Supplementary Materials

Supplementary materials can be found at https://www.mdpi.com/1422-0067/22/5/2481/s1, Figure S1: Morpho-physiological differences in two blueberry species. Figure S2: Volcano and MA plots of DEGs. Figure S3: DEGs in fatty acid degradation pathway map of alignment of *V. corymbosum* DEGs at 6-h heat stress to *Vitis vinifera* genome. Figure S4: Glycine, serine, and threonine metabolism pathway: alignment of *V. corymbosum* DEGs at 6-h heat stress to *V. vinifera* genome. Figure S5: Ascorbate and Aldarate metabolism pathway: alignment of *V. corymbosum* DEGs at 6-h heat stress to *V. vinifera* genome. Figure S6: Peroxisome pathway: alignment of *V. corymbosum* DEGs at 9-h heat stress to *V. vinifera* genome. Figure S7: Ribosome pathway: alignment of *V. darrowii* at 6-h heat stress to *V. vinifera* genome. Figure S8: Photosynthesis-antenna protein pathway: alignment of *V. darrowii* at 6-h heat stress to *V. vinifera* genome. Figure S9: Circadian-rhythm plant pathway: alignment of *V. darrowii* data at 9-h heat stress to *Arabidopsis thaliana* genome. Figure S10: Biosynthesis pathway activity by gene counts of *V. corymbosum* at 6-h and 9-h heat stress depicting up- and downregulation. Figure S11: Biosynthesis pathway activity by gene counts of *V. darrowii* at 6- and 9-h heat stress depicting up- and downregulation. Table S1: Top 10 and all the DEGs in *V. corymbosum* and *V. darrowii* at 6 and 9 h of heat stress. Table S2: Pathway enrichment analysis output from clusterProfiler using KEGG database for heat stressed DEGs aligned to genome *Vitis vinifera.* Table S3: Pathway enrichment analysis output from clusterProfiler using KEGG database for gene alignment to *Arabidopsis thaliana.* Table S4: DEGs in metabolic pathways of *V. corymbosum* at 6 hhs. Table S5: Details of the genes selected for RT-qPCR analysis.
