# Supplementary material for: Differential Morpho-Physiological and Transcriptomic Responses to Heat Stress in Two Blueberry Species
Source: Int J Mol Sci. 2021 Mar 1;22(5):2481. doi: 10.3390/ijms22052481 (PMC7957502; doi:10.3390/ijms22052481)
Supplement: Supplementary file 1 [file ijms-22-02481-s001.zip › Supplementary material/Callwood et al. Supplementary Figures.docx]

**Supplementary Figures**

Differential Morpho-Physiological and Transcriptomic Responses to Heat Stress in Two Blueberry Species

Jodi Callwood ^1^, Kalpalatha Melmaiee ^1,^*, Krishnanand P. Kulkarni ^1^, Amaranatha R. Vennapusa ^1^, Diarra Aicha ^1^, Michael Moore ^2^, Nicholi Vorsa ^3^, Purushothaman Natarajan ^4^, Umesh K. Reddy ^4^ and Sathya Elavarthi ^1^

**^1^** Department of Agriculture and Natural Resources, Delaware State University, Dover, DE-19901, USA; j.callwoo@gmail.com (J.C.); kkulkarni@desu.edu (K.P.K.); avennapusa@desu.edu (A.R.V.); daicha@desu.edu (D.A.); selavarthi@desu.edu (S.E.)

^2^ Optical Science Center for Applied Research (OSCAR), Delaware State University, Dover, DE-19901, USA; mmoore@desu.edu

^3^ Philip E. Marucci Center for Blueberry and Cranberry Research and Extension, Rutgers University, Chatsworth, NJ 08019, USA; vorsa@njaes.rutgers.edu

^4^ Department of Biology and Gus R. Douglass Institute, West Virginia State University Institute, WV 25112, USA; pnatarajan@wvstateu.edu (P.N.); ureddy@wvstateu.edu (U.K.R.)

***** Correspondence: kmelmaiee@desu.edu

| **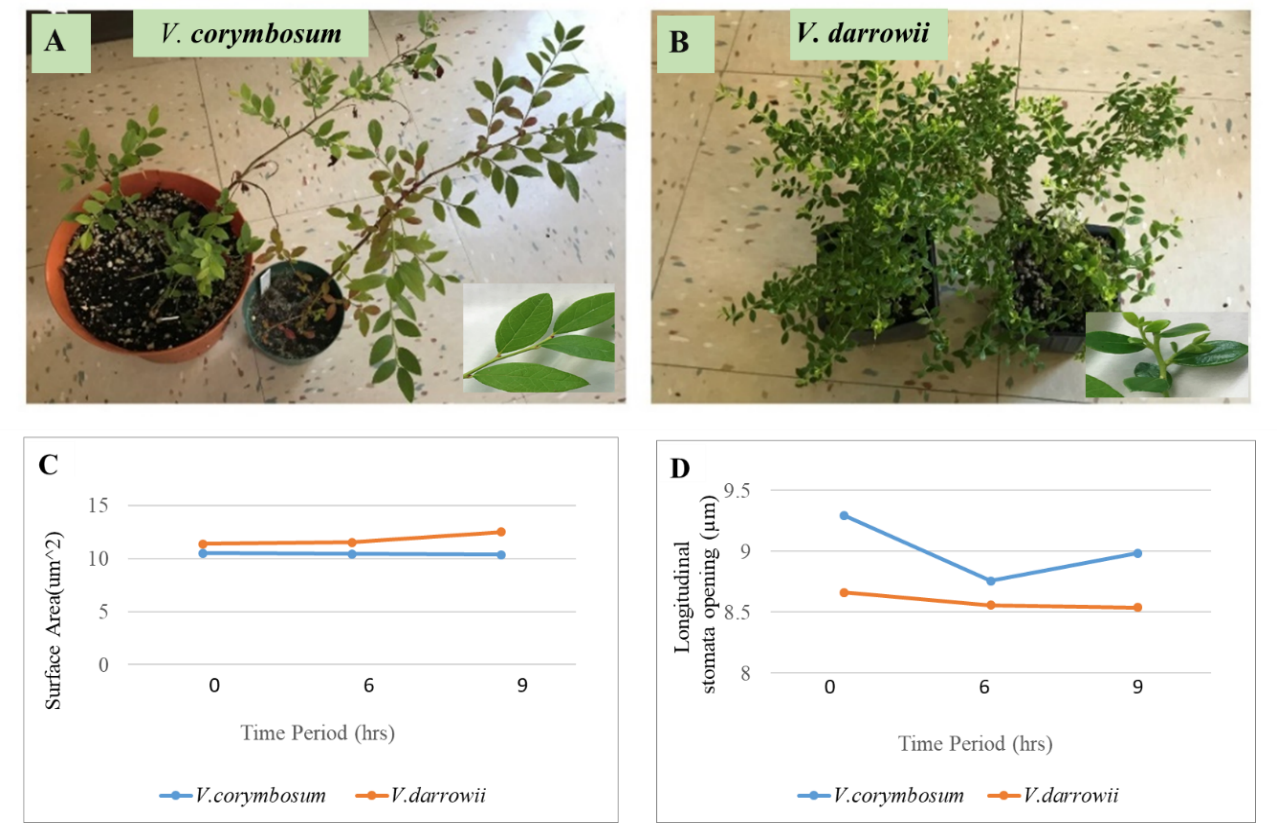** | |
| --- | --- |
| **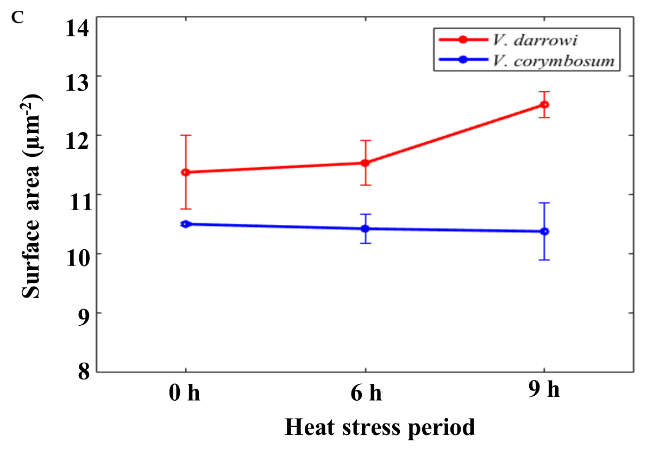** | **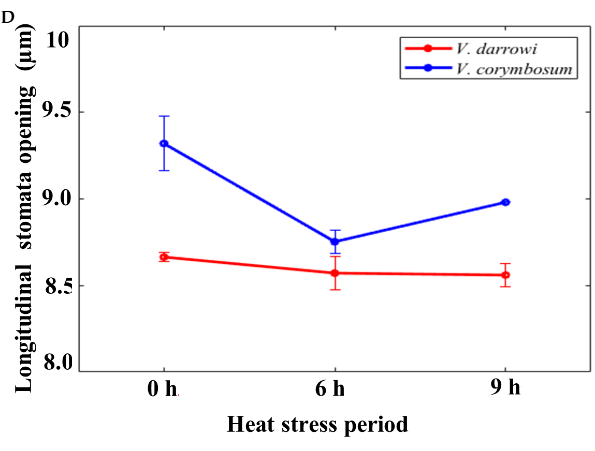** |

**Figure 1. Morpho-physiological differences in two blueberry species. A&B** Blueberry plant pictures depict the leaf size, pattern, and orientation. **A.** *V. corymbosum* **B.** *V. darrowii*. **C&D**: Assessment of heat stress effect on stomata and stomatal organelles by laser scanning confocal microscopy (LSCM) imaging analysis. **C.** Stomatal organelle surface area of auto-fluorescent signal measured using laser scanning confocal microscopy. **D.** Longitudinal distance between the stomatal pore cells (stomata opening).


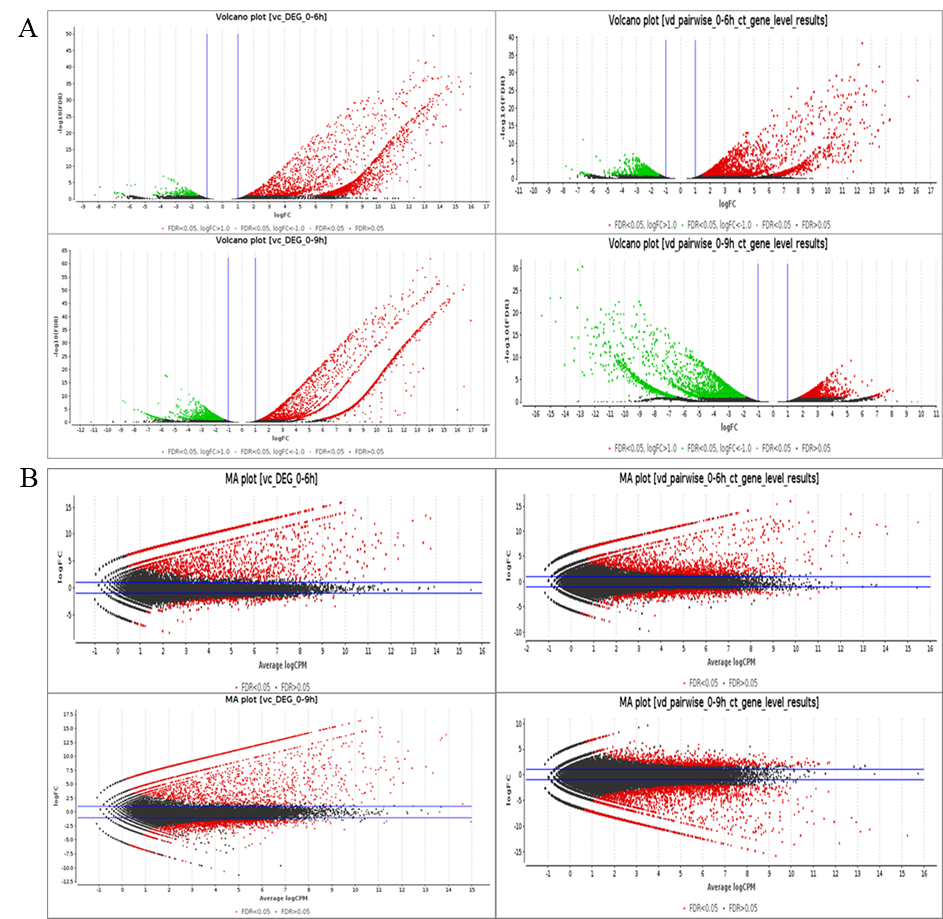


**Figure S2:** **Volcano and MA plots of DEGs**. **A.** Volcano plots of differentially expressed genes (DEGs) identified between *V. corymbosum*, and *V. darrowii* (p < 0.05 and log2 ratio ≥ 1). The upregulated genes are represented by red dots, and downregulated genes are represented by green dots. **B.** Log fold-change (M) over variance (A) plots (MA plots) of DEGs at 6 and 9 h of heat stress in *V. corymbosum* and *V. darrowii*.


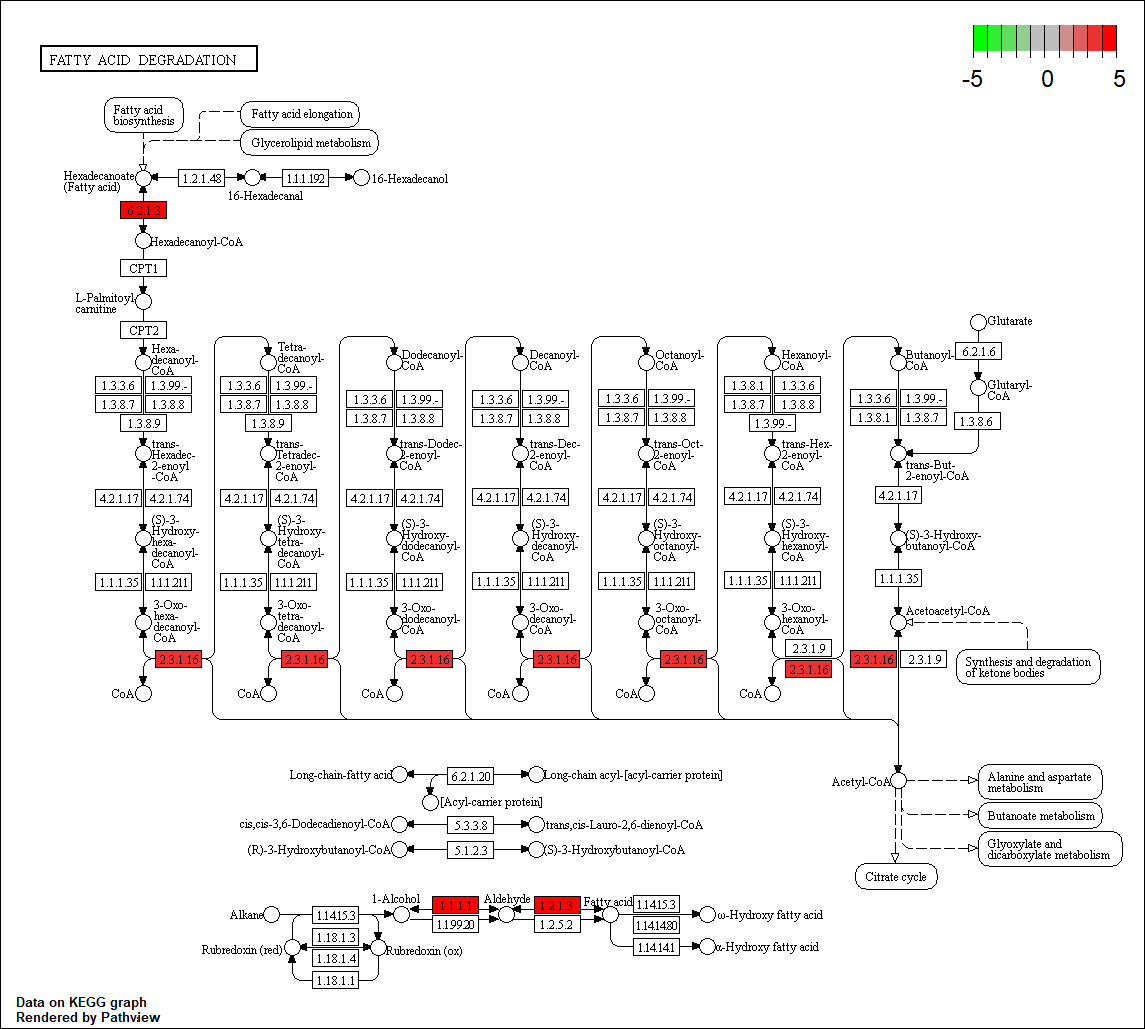
**Figure S3:** DEGs in fatty acid degradation pathway map of alignment of *V. corymbosum* DEGs at 6-h heat stress to *Vitis vinifera* genome.


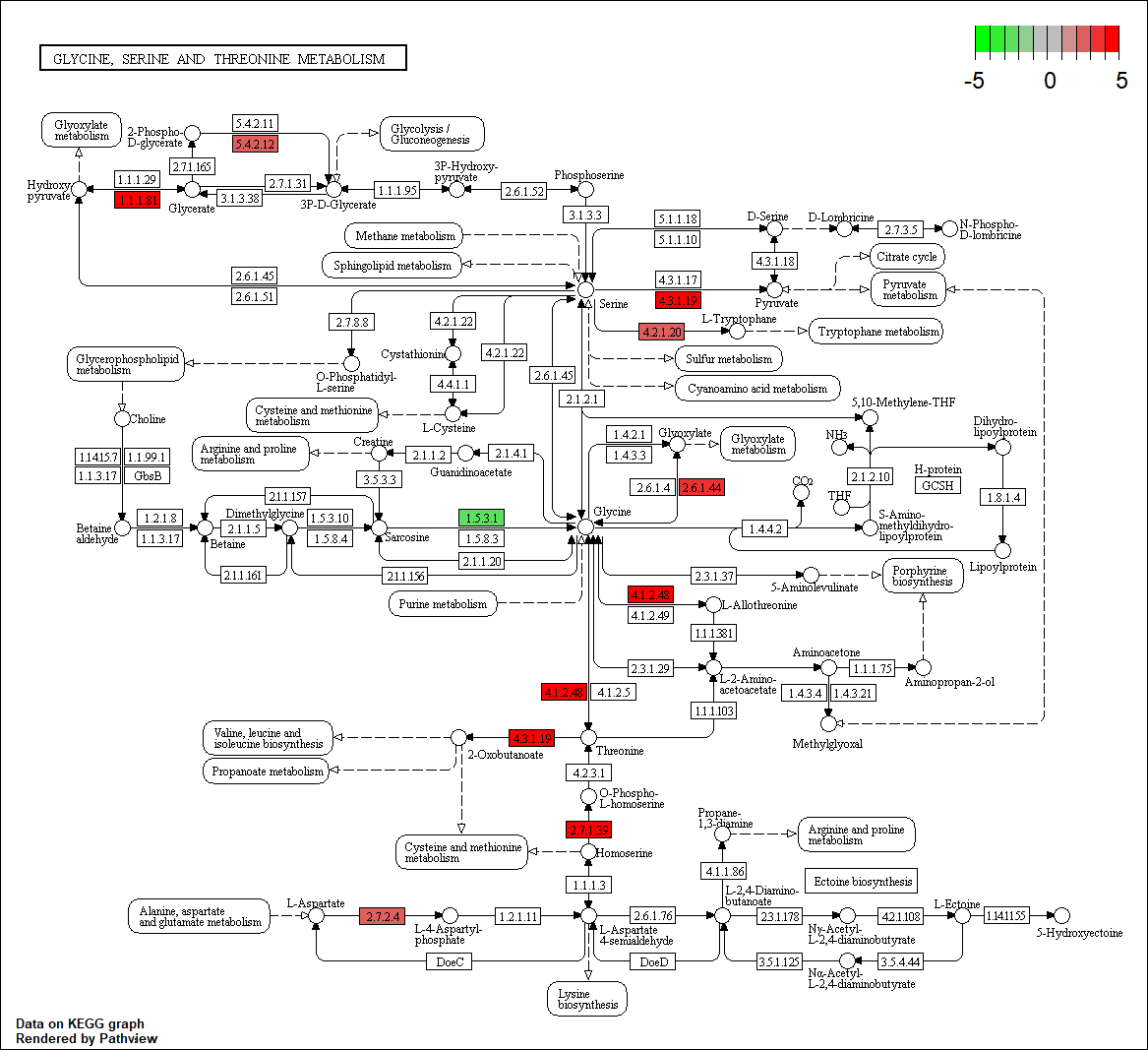


**Figure S4:** Glycine, serine, and threonine metabolism pathway: alignment of *V. corymbosum* DEGs at 6-h heat stress to *V. vinifera* genome. The colored box represents DEGs, and their degree of expression is depicted by color variations, with red the most upregulated and green the most downregulated.


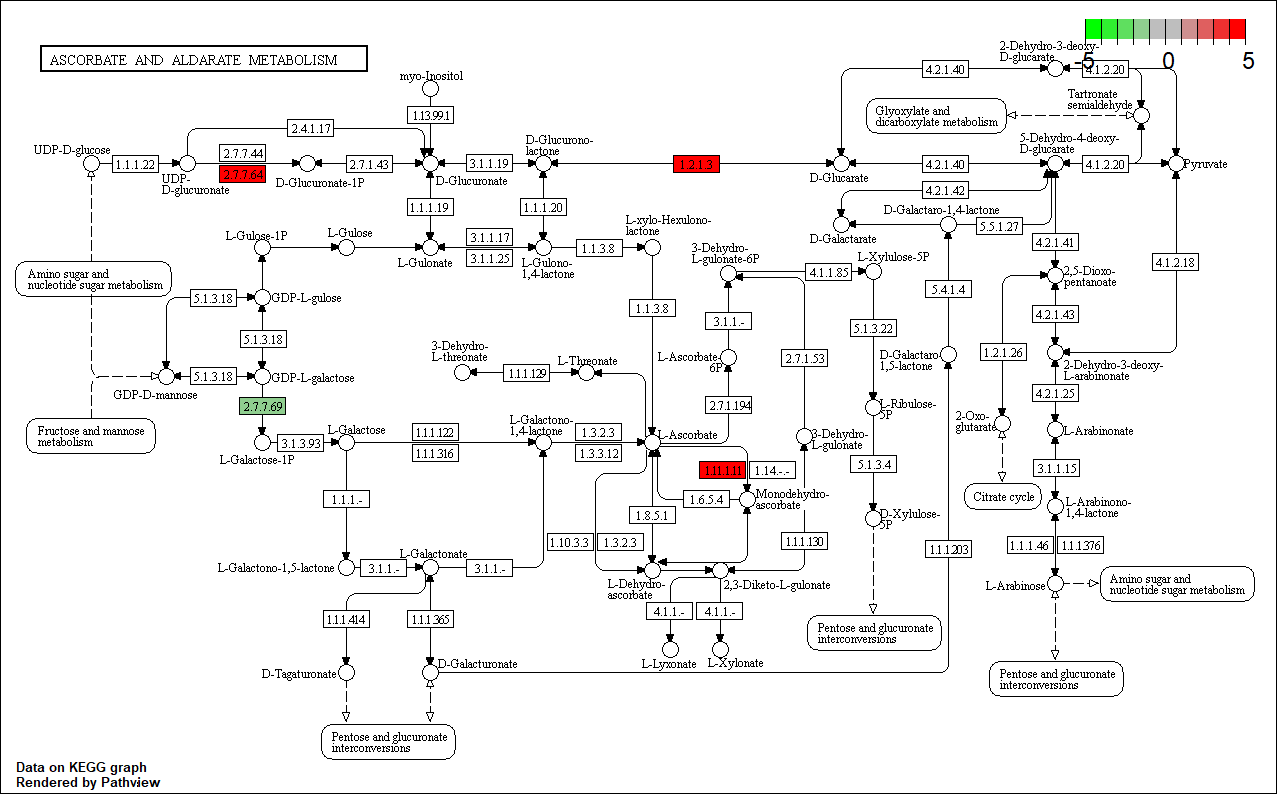


**Figure S5:** Ascorbate and aldarate metabolism pathway: alignment of *V. corymbosum* DEGs at 6-h heat stress to *V. vinifera* genome. The colored box represents DEGs, and their degree of expression is depicted by color variations, with red the most upregulated and green the most downregulated.


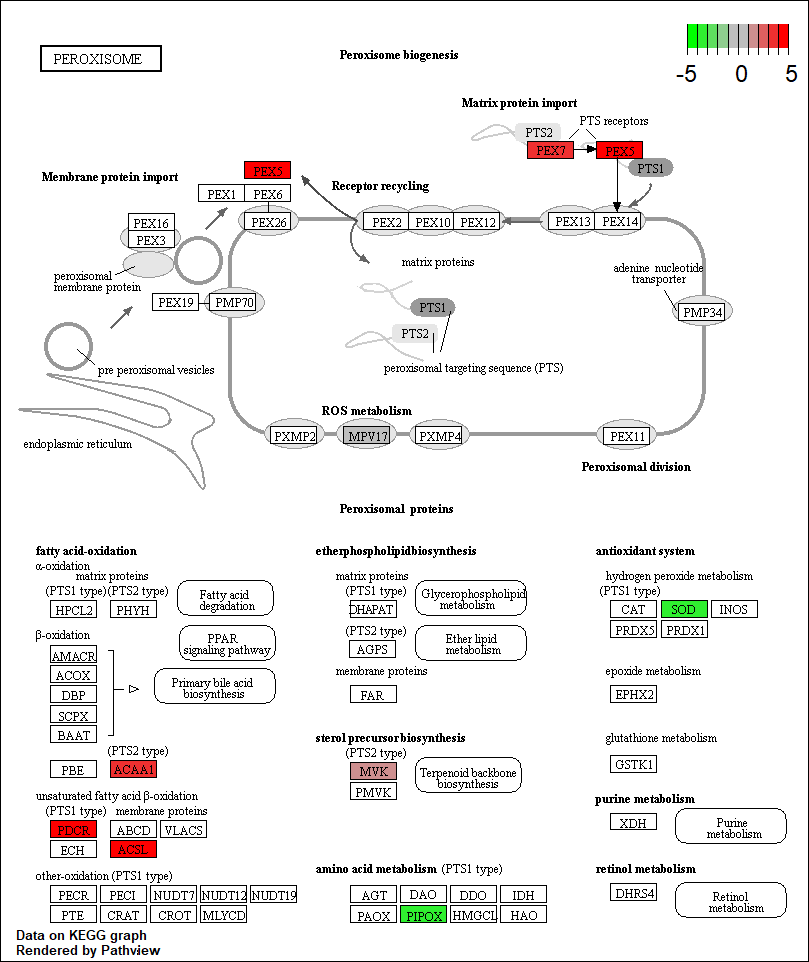


**Figure S6:** Peroxisome pathway: alignment of *V. corymbosum* DEGs at 9-h heat stress to *V. vinifera* genome. Upper diagram depicts peroxisomal membrane, and lower diagram depicts peroxisomal proteins divided into peroxisomal targeting signal (PTS) type one and two. The colored box represents DEGs, and their degree of expression is depicted by color variations, with red the most upregulated and green the most downregulated.


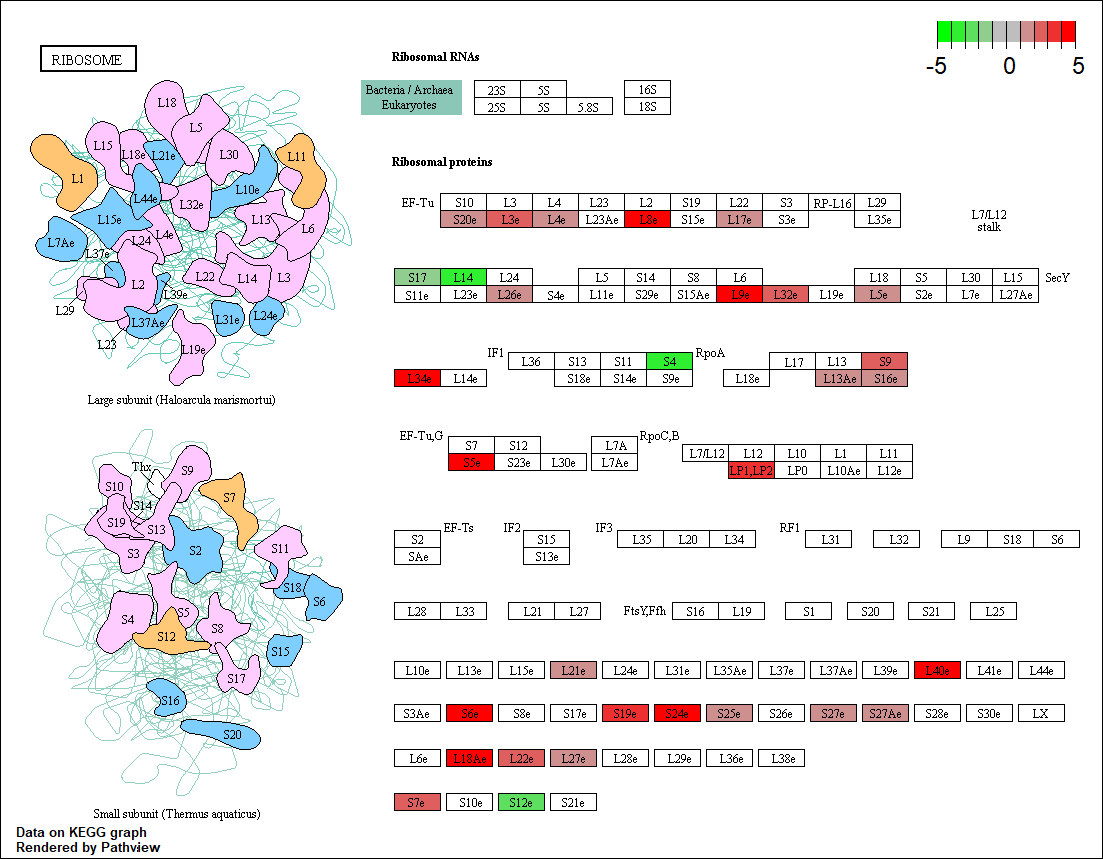
**Figure S7:** Ribosome pathway: alignment of *V. darrowii* at 6-h heat stress to *V. vinifera* genome. The colored box represents DEGs, and their degree of expression is depicted by color variations, with red the most upregulated and green the most downregulated. The L subunits correspond to the large ribosomal subunit (*Haloarcula marismortui*), and the S subunits correspond to the small ribosomal subunit (*Thermud aquaticus*).


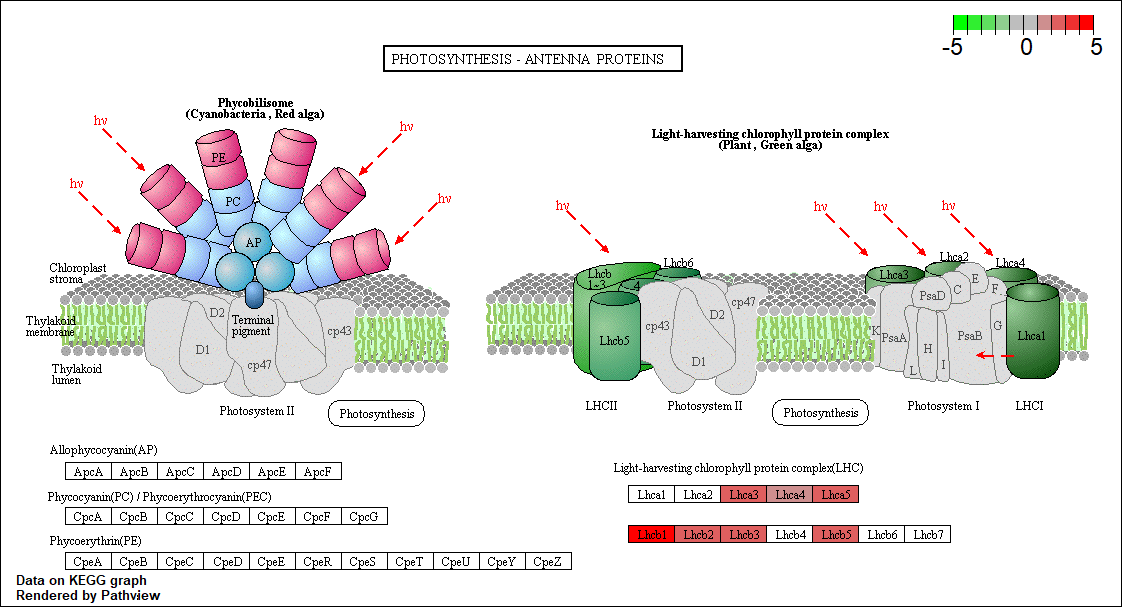


**Figure S8:** Photosynthesis-antenna protein pathway: alignment of *V. darrowii* at 6-h heat stress to *V. vinifera* genome. The colored box represents DEGs, and their degree of expression is depicted by color variations, with red the most upregulated and green the most downregulated.


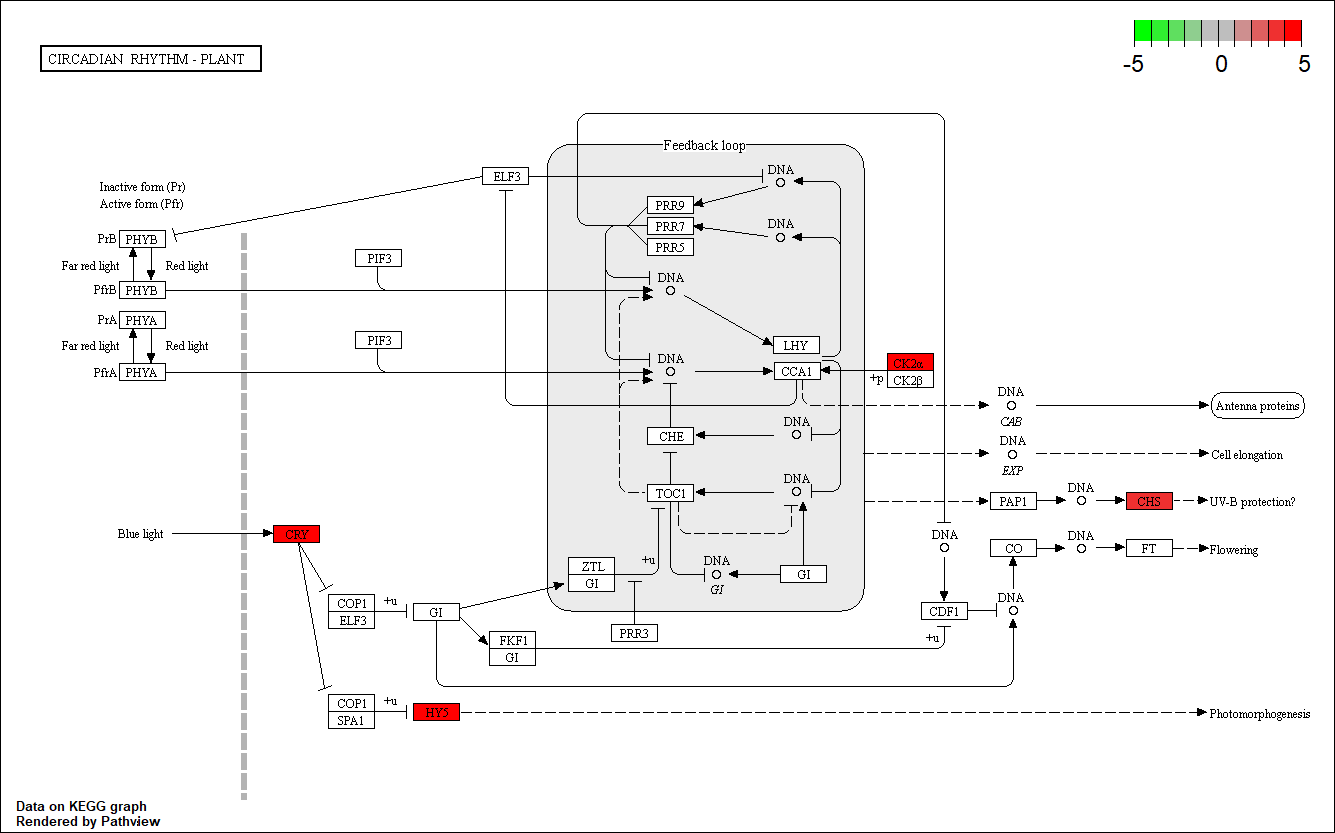


**Figure S9:** Circadian-rhythm plant pathway: alignment of *V. darrowii* data at 9-h heat stress to *Arabidopsis thaliana* genome. The colored box represents DEGs, and their degree of expression is depicted by color variations, with red the most upregulated and green the most downregulated.

**Figure S10:** Biosynthesis pathway activity by gene counts of *V. corymbosum* at 6-h and 9-h heat stress depicting up- and downregulation

**Figure S11:** Biosynthesis pathway activity by gene counts of *V. darrowii* at 6- and 9-h heat stress depicting up- and downregulation.
